# Headache in a Child with Pseudohypoparathyroidism: An Alarming Symptom Not to Miss

**DOI:** 10.1155/2020/8840082

**Published:** 2020-11-10

**Authors:** Sarah Wing-yiu Poon, Brian Hon-yin Chung, Anita Man-ching Tsang, Grace Wing-kit Poon

**Affiliations:** Department of Paediatrics and Adolescent Medicine, Queen Mary Hospital, LKS Faculty of Medicine, The University of Hong Kong, Pok Fu Lam, Hong Kong

## Abstract

**Background:**

While the endocrine manifestations of pseudohypoparathyroidism are well known, less is known about the associated brain and spine abnormalities. These abnormalities may present with nonspecific symptoms in the paediatric population, and lack of awareness to these uncommon manifestations of the disease may result in a delay in necessary intervention. *Case Presentation*. We herein present a case of known pseudohypoparathyroidism type 1a who presented initially with minor head injury. She later developed progressive worsening headache, increased irritability, and vomiting. Repeated imaging showed hydrocephalus and Chiari malformation type 1 necessitating emergency craniectomy.

**Conclusion:**

Growth hormone deficiency, a common manifestation of pseudohypoparathyroidism type 1a, results in underdevelopment of the posterior cranial fossa and may account for the higher incidence of Chiari malformation in this group of patients. Other associated neurological features reported in pseudohypoparathyroidism type 1a include spinal stenosis, syringomyelia, and craniosynostosis. While less commonly seen, awareness to these associations is important in order to optimize the multidisciplinary care to this group of patients.

## 1. Background

Pseudohypoparathyroidism (PHP) and related disorders comprise a spectrum of disorders characterized by parathyroid hormone (PTH) resistance. Based on different molecular abnormalities and clinical features, PHP can be divided into several subtypes. Pseudohypoparathyroidism type 1a (PHP1A) is caused by heterozygous inactivating mutations on the maternal allele of the guanine nucleotide-binding protein, alpha subunit (*GNAS*) gene. These mutations result in impaired G-protein coupled receptor signalling and end-organ resistance to PTH, thyrotropin-stimulating hormone (TSH), gonadotropins, and growth hormone-releasing hormone (GHRH) [1]. While it is known that PHP1A is associated with Albright hereditary osteodystrophy (AHO), less is known about the association with brain and spine abnormalities.

In this report, we present a case of known PHP1A who required emergency craniectomy as a result of hydrocephalus and Chiari malformation type 1 (CM-1). It is important to highlight this rare association such that prompt imaging can be performed in any child with PHP1A who presents with symptoms of raised intracranial pressure.

## 2. Case Presentation

**Figure 1:**
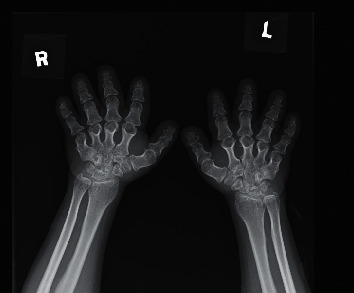
Patient's mother's X-ray hand demonstrating brachydactyly.

This girl was the first child born full term to a Chinese nonconsanguineous couple with body weight 1.73 kg (<3rd centile), body length 38 cm (<3rd centile), and head circumference 31 cm (<3rd centile). Apart from short stature (140 cm), her mother also has brachydactyly, spinal stenosis, and mild mental retardation (Figure 1). Antenatal checkup for this pregnancy showed short long bones, and the finding was confirmed on newborn assessment. Newborn screening also showed congenital hypothyroidism with elevated TSH, presence of normal thyroid gland but absent uptake on thyroid technetium scintiscan. She was thus started on thyroxine replacement since 2 months of age. She had rapid weight gain during infancy, and her weight-for-height rose to 97th centile at 5 months.

**Figure 2:**
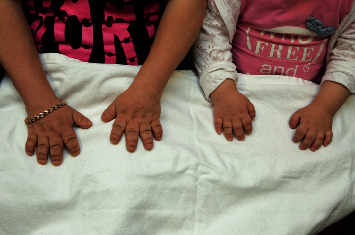
Patient's (left) and her mother's (right) hands demonstrating brachydactyly.

During the first year of life, her PTH was elevated (9.1–16 pmol/L, ref: 1.3–6.8 pmol/L), while her calcium and phosphate were normal. She started to develop hyperphosphataemia (2.81 mmol/L, ref: 1.20–1.80 mmol/L) and hypocalcaemia (1.75 mmol/L, ref: 2.17–2.51 mmol/L) since 14 months and was started on oral calcium gluconate and alfacalcidol. Physical examination showed round facies, acral shortening of the long bones, and brachydactyly (Figure 2). In view of clinical features of AHO and PTH resistance, PHP1A was suspected. Genetic analysis at 1.5 years of age confirmed the diagnosis of PHP1A with heterozygous mutation of the *GNAS* gene (*NM_001077488. c.C394G.p.(L132V*). Her mother was also found to have the same mutation.

**Figure 3:**
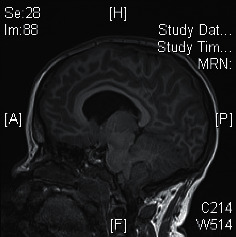
MRI brain showing hydrocephalus with obstruction at the craniocervical junction.

**Figure 4:**
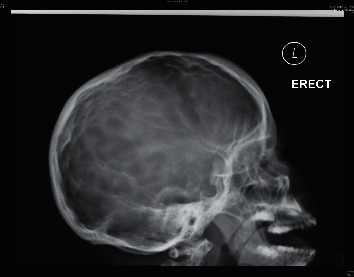
Skull X-ray showing copper beaten skull appearance.

Magnetic resonance imaging (MRI) of the brain was performed at 2 years of age for suspected central sleep apnoea, which revealed no abnormalities. At the age 4 years, she presented to the emergency department for minor head injury. Skull *X*-ray showed no skull fracture, and she was discharged. Six months later, she presented with increasing headache, irritability, and occasional vomiting. Upon admission, her vital signs were stable and neurological assessment was normal. Urgent MRI of the brain showed hydrocephalus with obstruction at the craniocervical junction. The posterior cranial fossa was small with severe crowding of the foramen magnum and inferior herniation of the cerebellar vermis causing cervicomedullary kinking (Figure 3). Emergency posterior fossa craniectomy for decompression was performed. On reviewing the skull X-ray taken 6 months prior to this hospital admission, generalized prominent gyri with copper beaten skull appearance was noted (Figure 4).

She had an uneventful postoperative course, and there was no recurrence of headache. Repeated MRI brain 5 months after operation showed interval improvement of her hydrocephalus and much reduced crowding at the foramen magnum. Upon follow-up, she was gaining height even though she was still below the 3rd centile and her body weight remained along the 75th centile. Clonidine and glucagon stimulation tests confirmed growth hormone deficiency (GHD), and she was started on growth hormone (GH) replacement therapy at 5 years of age.

Regular monitoring of her PTH, calcium, phosphate, and thyroid function showed satisfactory parameters. The girl has global developmental delay and is currently receiving extra training at a special school with satisfactory developmental progress.

## 3. Discussion and Conclusions

We hereby report a case of PHP1A who developed hydrocephalus with CM-1.

CM-1 is defined as caudal displacement of the cerebellar tonsils below the foramen magnum and can result in life-threatening complications as a result of cerebellar compression, spinal cord dysfunction, or central sleep apnoea.

In our case, lack of awareness to the association of PHP1A and CM-1 resulted in a delay in diagnosis on the initial skull X-ray and subsequent neurosurgical emergency. While endocrine defects in PHP1A are well known, nonendocrine features of PHP1A are less commonly described. Shoemaker et al. described the nonclassic features of PHP1A including various orthopedic complications and ear-nose-throat findings [2]. Specific dental and craniofacial features in patients with PHP1A and maternal *GNAS* mutation have also been described in a systematic analysis, with high rate of patients showing dental anomalies and alteration in craniofacial bone development [3]. Association of neurologic dysfunction with PHP1A is not well appreciated. Two cases on the association of PHP1A and CM-1 have been reported in the literature [4, 5]. Martínez-Lage et al. reported a girl with PHP1A who also complained of mild headache, with imaging showing cerebellar tonsillar descent. In contrast to our case, tonsillar descent regressed with time [4]. In another report by Kashani et al., a younger patient with PHP1A had MRI brain performed for evaluation of developmental delay at 34 months, which revealed CM-1 with hydrocephalus requiring neurosurgical intervention. Similar to our patient, he had a normal MRI brain earlier in life [5]. These cases demonstrated that CM-1 may develop with time presenting with nonspecific symptoms, and hence, a low threshold in performing brain imaging should be adopted.

Multiple theories have been proposed for Chiari malformation (CM), one of which is the small posterior fossa theory. In our patient, MRI brain also showed a small posterior fossa volume with reduced craniocaudal height and crowding of the foramen magnum. The small posterior fossa theory was initially described by Marin-Padilla in 1981, where it was hypothesized that a mismatch between the size of the posterior fossa and its content leads to neural element compression and caudal herniation [6]. As such, GHD may also contribute to the underdevelopment of the posterior fossa. GH has an important role on cartilage growth, and a study showed that GH excess or deficiency may affect cartilaginous growth loci at cranial base [7]. In fact, Hamilton et al. performed MRI on 35 patients with GHD, and up to 20% of them have Chiari malformations [8]. With impaired responsiveness of somatotrophs to GHRH, PHP1A patients are thus at higher risk of CM due to GHD. Our patient, who was confirmed to have GHD at 5 years of age, had a normal MRI brain at 2 years of age. This demonstrated that underdevelopment of the posterior fossa might evolve with time and normal brain imaging earlier in life might not rule out the possibility of neurological abnormalities as the child grows.

Other associated neurological features reported in PHP1A include spinal stenosis, syringomyelia, and craniosynostosis. Our patient's mother suffers from chronic low back pain and lower limb numbness as a result of diffuse spinal stenosis. This highlights the importance to attend to the nonendocrine manifestation of the disease, as well as the need to offer multidisciplinary evaluation and therapy in PHP1A patients at all stages of life.

## Data Availability

The datasets used during the current study are available from the corresponding author on reasonable request.

## Abbreviations

PHP: Pseudohypoparathyroidism
PHP1A: Pseudohypoparathyroidism type 1a
PTH: Parathyroid hormone
GNAS: Guanine nucleotide-binding protein, alpha subunit
TSH: Thyrotropin-stimulating hormone
GHRH: Growth hormone-releasing hormone
AHO: Albright hereditary osteodystrophy
CM-1: Chiari malformation type 1
CM: Chiari malformation
MRI: Magnetic resonance imaging
GHD: Growth hormone deficiency
GH: Growth hormone.
